# *In Vitro* and *In Vivo* Antihypertensive Effect of Milk Fermented with Different Strains of Common Starter Lactic Acid Bacteria

**DOI:** 10.3390/nu14245357

**Published:** 2022-12-16

**Authors:** Olga A. Glazunova, Konstantin V. Moiseenko, Olga S. Savinova, Tatyana V. Fedorova

**Affiliations:** A.N. Bach Institute of Biochemistry, Research Center of Biotechnology, Russian Academy of Sciences, 119071 Moscow, Russia

**Keywords:** lactic acid bacteria, milk fermentation, angiotensin-converting enzyme inhibition, fatty acids, nutritional indices, spontaneously hypertensive rats, blood cholesterol

## Abstract

Currently, functional dairy products pave a promising way for the prophylaxis of essential hypertension, and the search for new strains capable of producing such products is a constant challenge for scientists around the world. In this study, the antihypertensive properties of milk fermented with several strains of traditional yogurt starters (*Lactobacillus delbrueckii* strains Lb100 and Lb200; *Lactococcus lactis* strains dlA, AM1 and MA1; *Streptococcus thermophilus* strains 159 and 16t) and one strain of non-conventional probiotic starter (*Lacticaseibacillus paracasei* ABK) were assessed. The *in vitro* assessment using angiotensin-converting enzyme inhibition assay was performed for all fermentation products, and the best performed products were tested *in vivo* using Spontaneously Hypertensive Rat (SHR) animal model. In addition, for the best performed products the fatty acid (FA) composition and FA-related nutritional indices were determined. As a result, the milk fermented with two strains (*Lb. delbrueckii* LB100 and *Lc. lactis* AM1) demonstrated significant antihypertensive effect during both *in vitro* and *in vivo* experiments. Moreover, the milk fermented with *Lb. delbrueckii* Lb100 demonstrated significantly better FA-related nutritional indexes and lowered total cholesterol in SHRs upon regular consumption. The obtained results can be used in the future to develop new starter cultures producing effective functional antihypertensive dairy products.

## 1. Introduction

According to the recently published study, which analyzed the prevalence of hypertension and progress in its detection, treatment and control from 1990 to 2019 for 200 countries and territories, the number of people with hypertension has nearly doubled in the past 30 years—from 650 million to 1.28 billion worldwide [1]. The experts of the World Health Organization (WHO, Geneva, Switzerland) constantly emphasize that high blood pressure significantly increases the risk of cardiovascular diseases, as well as diseases of the brain and kidneys. Tens of millions of people die every year due to hypertension-related causes [2].

Currently, the use of inhibitors of the renin-angiotensin-aldosterone system (RAAS) is recommended first-line evidence-based therapy for patients with arterial hypertension [3,4,5]. The RAAS is one of the evolutionary oldest hormonal systems that serve primarily to balance blood pressure and electrolyte homeostasis [6]. In short, the functioning of the RAAS can be described as follows [7]: Upon a reduction in blood volume or a drop in blood pressure, the kidneys start to secrete a specific enzyme, renin, into the bloodstream. In the bloodstream, renin converts 12 aa peptide angiotensinogen (secreted by liver) into decapeptide angiotensin I. The angiotensin I is further converted by the angiotensin-converting enzyme (ACE) locating on the surface of vascular endothelial cells (mainly the lungs) into octopeptide angiotensin II. The action of angiotensin II causes narrowing of the blood vessels and increases the reabsorption of water into the blood which in turn causes an increase in blood pressure.

The most well-known RAAS inhibitors are synthetic drugs such as captopril, enalapril and perindopril, which tend to inhibit ACE activity leading to the relaxation of blood vessels and lowering of blood pressure [8]. These synthetic drugs are widely used in medicine for the treatment of hypertension; however, their unpleasant side effects (e.g., abnormal taste, skin rashes and coughing) and hepatotoxicity have currently prompted the development of natural, safe, and novel alternatives such as “ACE inhibiting (ACE-I) foods” [9]. In their composition ACE-I foods contain specific peptides that can interact with an active site of ACE and concurrently inhibit conversion of angiotensin I into angiotensin II [10]. However, it should be especially emphasized that at the present state of development consumption of the ACE-I food can be regarded only as a prophylaxis but not as a treatment of hypertension [11].

Currently, one of the best studied ACE-I foods are dairy products produced by the fermentation with lactic acid bacteria (LAB) [12,13]. During the fermentation, proteolytic system of LAB generates small peptides from milk’s proteins (mainly α, β and κ-caseins), which may have various beneficial properties including ACE-I [14]. However, the proteolytic systems in various LAB are extremely diverse and vary not only between different LAB species but also between different strains of the same species [15,16,17]. Consequently, the selection of conventional starter strains producing fermented milk with higher ACE-I properties or the inclusion of non-conventional starter strains into the fermentation in order to enrich final product with ACE-I peptides is extremely important and active direction of research [18].

Analyzing the enrichment of fermented dairy products with ACE-I properties, it is especially worth paying attention to the fact that in some cases, there is no correlation between the ACE-I activity of fermented milk measured *in vitro* and its hypotensive effect *in vivo* [19]. On the one hand, the *in vivo* antihypertensive effect can be less than expected due to the fact that peptides in the gastrointestinal tract may be further degraded during digestion, which may lead to a decrease in their bioavailability and beneficial effects [20]. On the other hand, besides ACE-I peptides fermented milk products contain LAB cells and their metabolites that upon ingestion can exert an additional hypotensive effect via induction of eubiosis, reduction in oxidative stress and improvement of endothelial function [21,22,23,24,25]. In addition, the fermentation of milk changes composition of its matrix components (e.g., calcium, peptides, phosphorus, amino and fatty acids) and drastically influences health outcomes of its consumption [26]. Hence, it is of paramount importance not only test ACE-I of products *in vitro*, but also to substantiate their antihypertensive action *in vivo* using suitable animal models.

In this article, several strains of traditional yogurt starters (*Lactobacillus delbrueckii*, *Lactococcus lactis* and *Streptococcus thermophilus*) and one strain of non-conventional starter (*Lacticaseibacillus paracasei*) were tested on their ability to produce fermented dairy products with ACE-I properties. The milk fermented by the best performed strains was further analyzed and its fatty acid (FA) content as well as FA-related nutritional indices were determined. In addition, the antihypertensive properties of the fermented milks were assessed *in vivo* using Spontaneously Hypertensive Rat (SHR) animal model.

## 2. Materials and Methods

### 2.1. Strains and Cultivation Conditions

The strains of LAB were obtained from the Collection of the All-Russian Research Institute of the Dairy Industry (VNIMI, Moscow, Russia), where they were kept at −80 °C in skim milk containing 10% (*v*/*v*) glycerol. For each strain, the GeneBank accession of the 16S rRNA sequence as well as optimal growth temperature used during all cultivations are presented in Table 1.

Upon reception, stock cultures of *Lactobacillus* strains were streaked on de Man, Rogosa and Sharpe (MRS) medium (HiMedia Laboratories, Mumbai, India) and grown overnight at the optimal temperature (Table 1) under anaerobic conditions using Anaero Bag System 24 (HiMedia Laboratories, Mumbai, India). Stock cultures of *Lactococcus* and *Streptococcus* strains were streaked on M17 medium (HiMedia Laboratories, Mumbai, India) and grown overnight at the optimal temperature (Table 1) under aerobic and anaerobic conditions, respectively. To obtain a preculture, the *Lactobacillus* colonies were inoculated in MRS broth and *Lactococcus* and *Streptococcus* colonies in M17 broth and grown at the optimal temperature (Table 1) to the 10^6^–10^7^ CFU∙mL^−1^, depending on the strain. To obtain a working culture, commercial skim milk powder grade “Standard” (Complimilk, Slutsk cheese-making plant, Slutsk, Belarus) was reconstituted (12%, *w*/*v*), sterilized (110 °C, 10 min), cooled to approximately 30 °C, inoculated with the preculture (3%, *v*/*v*) and incubated overnight at the optimal growth temperature (Table 1).

For milk fermentation, one liter of the reconstituted skim milk (RSM), prepared as described above, was pasteurized (80–85 °C for 30 min), cooled to approximately 30 °C and inoculated with the working culture (1%, *v*/*v*). The fermentation was performed at the optimal growth temperature (Table 1). Samples were taken for analysis at 0, 6, 16, 24, 48, and 72 h of fermentation. For all strains but *Lb. paracasei* ABK, the samples from the 16 h of fermentation were used in the animal experiments, for which they were cooled and stored at 4 °C until use. For *Lb. paracasei* ABK, the samples from the 48 h of fermentation were used in the animal experiments.

### 2.2. Proteolytic, Antioxidant, and Angiotensin-I-Converting Enzyme Inhibitory Activities

The fermented milk samples with pH 4.6 and below were centrifuged at 3000× *g* for 15 min at 4 °C (Eppendorf centrifuge 5430 R, Hamburg, Germany). If the pH of the samples was higher than 4.6, the samples were preliminarily titrated with trichloroacetic acid (TCA, 0.75%, *w*/*v*) to pH 4.6. The supernatant was stored at −20 °C in 2 mL-aliquots. Prior to use, the supernatant was thawed at 4 °C, centrifuged at 10,000× *g* for 3 min at room temperature, and filtered through a 0.45 µm syringe filter with hydrophilic membrane (Merk Millipore, Darmstadt, Germany).

The proteolytic activity in the obtained supernatant was measured using the TNBS (2,4,6-trinitrobenzenesulfonic acid) method according to Adler-Nissen [27]. The 0.25 mL of the diluted in SDS (1% *w*/*v*) sample was mixed with 2 mL of sodium phosphate buffer (0.2 M, pH 8.2). Then, 2 mL of TNBS (Sigma-Aldrich, St. Louis, MO, USA) reagent (0.1% *w*/*v* in water) was added. Test tubes were mixed and incubated at 50 °C for 60 min, and the reaction was stopped by the addition of 4 mL of 0.1 N HCl. Absorbance was measured at 340 nm using a Synergy 2 microplate photometer–fluorimeter (BioTek, Winooski, VT, USA). A calibration curve was prepared using L-leucine (L-Leu) as a standard (0.1–2.0 mM). The proteolytic activity was expressed as L-Leu molar equivalents, mM (Leu).

The antioxidant activity in the obtained supernatant was determined by the oxygen radical absorbance capacity fluorescence method (ORAC) with generation of the peroxyl radical according to Ou, Hampsch-Woodill, and Prior [28] with slight modifications as described in Nikolaev et al. [29]. The antioxidant capacity of samples against peroxyl radicals was expressed as an amount of Trolox molar equivalents, mM (Trolox).

The ACE-I activity in the obtained supernatant was determined in terms of half maximal inhibitory concentration (IC_50_), as described in Torkova et al. [30]. ACE activity was measured using o-Aminobenzoyl-Phe-Arg-Lys(dinitrophenyl)-Pro (Sigma-Aldrich, St. Louis, MO, USA) as a substrate with internal fluorescence quenching. The measurements were carried out on a Synergy 2 microplate photometer–fluorometer (BioTek, Winooski, VT, USA).

### 2.3. Fatty Acid Profile of Fermented Milk

The FAs extraction from the samples of fermented milk was performed according to the Folch method. After the extraction, FAs were derivatizedusing 3 M methanolic HCl (Supelco, Bellefonte, PA, USA), according to the manufacturer’s protocol. Derivatized FAs were separated using GC 2010 chromatograph (Shimadzu, Kyoto, Japan) equipped with an MDN-5 column (30 m × 0.25 mm; Bellefonte, PA, USA) and analyzed using a mass detector GCMS-QP 2010 in the regime of temperature gradient. The PUFA-2 (Supelco, Bellefonte, PA, USA) kit was used as a standard. The identification of FAs was carried out as described in Moiseenko et al. [31]. The relative intensities (further relative abundances) of FA were obtained by normalization on the total intensity of the assigned peaks. All experiments were performed in triplicate.

The FA-related nutritional indices were calculated according to Chen et al. [32]:

PUFA/SFA = ∑PUFA/∑SFA(1)

IA = [C12:0 + (4 × C14:0) + C16:0]/[∑MUFA + ∑PUFA](2)

HPI = [∑MUFA + ∑PUFA]/[C12:0 + (4 × C14:0) + C16:0](3)

IT = [C14:0 +C16:0 + C18:0]/[0.5 × (∑MUFA + ∑PUFA{*n* − 6})] (4)

HH = [C18:1 + ∑PUFA]/[C12:0 + C14:0 + C16:0](5)

UI = 1 × (%monoenoics) + 2 × (%dienoics) + 3 × (%trienoics) + 4 × (%tetraenoics)(6)

where SFA stands for saturated fatty acids; MUFA—monounsaturated fatty acids; PUFA—polyunsaturated fatty acids; PUFA/SFA—ratio of total PUFA to total SFA; IA—index of atherogenicity; HPI—health-promoting index (which is the reciprocal of IA and mainly used in research on dairy products); IT—index of thrombogenicity; HH—hypocholesterolemic/hypercholesterolemic ratio; UI—unsaturation index.

### 2.4. Spontaneously Hypertensive Rat (SHR) Animal Model

The hypotensive effect of selected fermented milks was studied using SHR animal model. A total of thirty male SHRs (27 weeks old; 286 ± 15 g body weight; 165 ± 15 mmHg systolic (Psyst) and 107 ± 18 mmHg diastolic (Pdiast) blood pressures, respectively) were obtained from Puschino Kennel of Laboratory Animals (Pushchino, Russia).

For the experiment, the SHRs were randomized into six groups of five animals each. In addition to the standard ration (Laboratormkorm, Moscow, Russia), the groups *ad libitum* received: (1) distilled water (the intact group); (2) 20 mL per day per animal of RSM (the control group); (3)–(6) 20 mL per day per animal of RSM, fermented with *Lb. delbrueckii* Lb100, *Lb. paracasei* ABK, *Lc. lactis* AM1, and *Str. thermophilus* 159, respectively. Each SHR group was housed in a separate cage at 22 ± 1 °C and 12 h:12 h light-dark cycles. The experiment was carried out for four weeks, and all the cages were cleaned from the remaining feed residues 12 h before the end of the experiment. At the end of the experiment, the SHRs were euthanized in a carbon dioxide chamber (VetTech, Congleton, UK). The samples of blood serum and aorta were collected and stored at −80 °C for further analysis. The blood serum was obtained by centrifugation of blood using Eppendorf 5702 R centrifuge (Eppendorf, Hamburg, Germany) at 4 °C and 2000× *g* for 10 min.

### 2.5. Measurements of Blood Pressure

The Psyst and Pdiast of SHRs were measured with a CODA Monitor Rat-Cuff KIT (Kent Scientific, Torrington, CT, USA) using a tail-cuff. At least ten measurement cycles were performed for each animal, and the results were averaged.

### 2.6. Measurments of Biochemical Parameters

The ACE activity in the aorta and blood serum samples was measured as described in Section 2.2 but without adding of ACE in the reaction mixture. Prior to measurements the aorta samples were homogenized with Silent Crusher M homogenizer (Heidolph, Schwabach, Germany) in the buffer used for ACE activity measurements.

To determine the enzymatic activities in blood serum of alanine aminotransferase (ALT), aspartic aminotransferase (AST) and lactate dehydrogenase (LDH), as well as to measure concentrations of total cholesterol (Chl), triglycerides (TG), cholesterol in high-density lipoproteins (HDLc), diagnostic kits for clinical chemistry (High Technology Inc., Attleboro Falls, MA, USA) were used. The analysis was performed on a BioChem FC-360 automatic chemistry analyzer (High Technology Inc., Attleboro Falls, MA, USA). The cholesterol concentration in low-density lipoproteins (FR-LDLc) was calculated according to the Friedewald’s equation [33]:

FR-LDLc = Chl − HDLc − TG/2.2(7)

The analysis of antioxidant capacity of blood serum was performed using trolox equivalent antioxidant capacity (TEAC) assay as described in Kruchinin et al. [34]. The antioxidant capacity of samples against ABTS radical was expressed as an amount of Trolox molar equivalents, mM (Trolox). Lipid peroxidation in blood serum samples was quantified by 2-thiobarbituric acid reactive substance (TBARS) assay according to method of Jentzsch et al. [35] with minor modifications as described in Kruchinin et al. [34]. The results were expressed as an amount of malondialdehyde (MDA) molar equivalents, µM (MDA).

### 2.7. Statistical Analysis

All statistical comparisons were firstly performed using one-way ANOVA omnibus *F*-Test. When a significant (*p* < 0.05) value of *F*-statistics was found, differences between means were evaluated using Tukey’s HSD (honestly significant difference) multiple comparison test (*p* < 0.05).

## 3. Results

### 3.1. The Fermentation Perfomance of Different Strains of Lactic Acid Bacteria (LAB): Selection of the Most Promissing, in Terms of the In Vitro Antioxidant and Antihypertensive Properties of the Fermented Milk, LAB Strains

**Figure 1:**
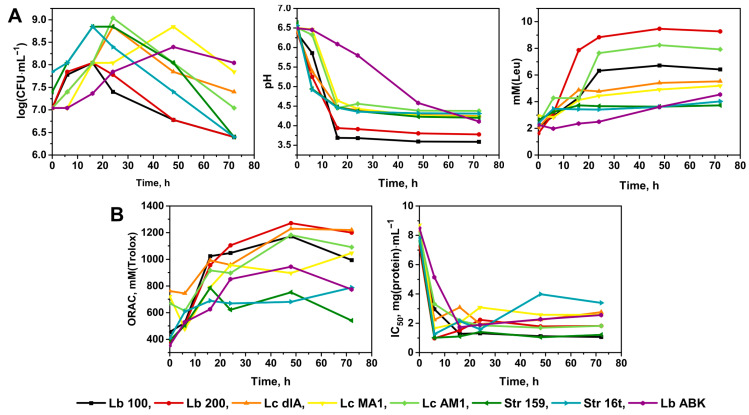
(**A**)—The general fermentation parameters of the screened strains of lactic acid bacteria; (**B**)—The development of antioxidant and ACE-I activities in the milk fermented by the screened strains of LAB. Strains of *Lactobacillus delbrueckii* and *Lacticaseibacillus paracasei* are abbreviated as Lb; strains of *Lactococcus lactis* and *Streptococcus thermophilus* are abbreviated as Lc and Str, respectively. For all datapoints the standard error from three biological replicas did not exceed 5%.

For the original screening, seven traditional yogurt starter strains of *Lb. delbrueckii*, *Lc. lactis* and *Str. thermophilus* and one non-conventional starter strain of *Lb. paracasei* were chosen. The general fermentation parameters such as: the dynamic of changes for the viable cell count (i.e., CFU) during the fermentation; the development of the pH in the fermented milk; and the degree of proteolysis of the fermented milk—are presented in Figure 1A. The dynamic of changes for the antioxidant and ACE-I activities of the fermented milk are presented in Figure 1B.

In terms of the general fermentation parameters (Figure 1A), the maximally attainable viable cell count was observed at 16–24 h of fermentation for all studied strains, after which it was steadily decreasing until 72 h. The exceptions were *Lc. lactis* MA1 and *Lb. paracasei* ABK; demonstrating significantly lower growth rate and a prominent lag-phase, these strains achieved the maximally attainable viable cell count only at 48 h of fermentation. For all strains of *Str. thermophilus* and *Lc. lactis* the maximally attainable viable cell count comprised 7.0 × 10^8^–1.1 × 10^9^ CFU·mL^−1^, and for both *Lb. delbrueckii* strains it comprised 1.1 × 10^8^ CFU·mL^−1^. For all *Str. thermophilus* and *Lc. lactis* strains, the pH values reached its minimum of approximately 4.5 after 16 h of fermentation. Similarly, for both *Lb. bulgaricus* strains, the minimal pH of approximately 3.7–3.9 was detected after 16 h of fermentation. The *Lb. paracasei* ABK demonstrated the slowest rate of pH decrease reaching the pH of 4.5 at approximately 48 h of fermentation. For all studied strains, the observed degrees of milk proteolysis during the first 16 h of fermentation were comparable. However, the final degrees of proteolysis, achieved after 24 h of fermentation, appeared to be strain specific. The exceptions were two strains of *Str. thermophilus* and two strains of *Lc. lactis* (dlA and MA1) for which the final degrees of proteolysis were similar.

For the antioxidant activity (Figure 1B), its development was comparable for all studied strains during the first 16 h of milk fermentation. After which, its value increased in a similar manner for almost all studied strains reaching approximately 1150 mM (Trolox) at the end of fermentation. As an exception, for both strains of *Str. thermophilus* the antioxidant activity did not change after 16 h of fermentation remaining at the same level of 700 mM (Trolox). It should be noted, that both strains of *Str. thermophilus* produced the smallest degree of milk proteolysis in the current experiment (Figure 1A). Previously we already discussed, that strains’ proteolytic activity generally correlates with antioxidant activity of milk fermented by them [36]. The main reason for this correlation is not very stringent requirements which peptides must meet in order to possess reasonable antioxidant activity [37,38,39].

In contrast to the antioxidant activity, the dynamic of changes for ACE-I activity does not always correlate with the degree of milk proteolysis, since ACE-I peptides are very sequence-specific [39]. In our study, *Str. thermophilus* strains having similar values of proteolytic activity demonstrated different ACE-I activity. On the contrary, while *Lb. delbrueckii* Lb100 had lower proteolytic activity than *Lb. delbrueckii* Lb200, it demonstrated higher ACE-I activity.

Among all studied strains of *Lb. bulgaricus* the highest ACE-I activity (i.e., the lowest IC_50_ value) was demonstrated by the milk fermented with *Lb. bulgaricus* Lb100—IC_50_ values of 1–1.5 mg·mL^−1^. Among the strains of *Str. thermophilus* the highest ACE-I activity was demonstrated by the milk fermented with *Str. thermophilus* 159—IC_50_ values of 1–1.5 mg·mL^−1^, similar with *Lb. bulgaricus* Lb100. Among the strains of *Lc. lactis* the higher ACE-I activity was demonstrated by the milk fermented with *L. lactis* AM1—IC_50_ values of 1.7–2.0 mg·mL^−1^. The IC_50_ value of the milk fermented with *Lb. paracasei* ABK comprised 2.1–2.5 mg·mL^−1^. Consequently, the milk fermented with *Lb. bulgaricus* Lb100, *Str. thermophilus* 159, *Lc. lactis* AM1 and *Lb. paracasei* ABK were chosen for further investigations.

### 3.2. Profile of Fatty Acids (FA) and FA Nutritional Indices for the Milk Fermented by the Selected LAB Strains

The qualitative composition and relative abundances (percentage from total FA) of FA for the milk fermented by the chosen LAB strains are shown in Table 2. In total, in all fermentation products, 38 different FA were detected, 18 of which belonged to the group of saturated fatty acids (SFA), seven to monounsaturated fatty acids (MUFA), three to polyunsaturated fatty acids (PUFA, two “*n*-6” and one “*n*-3”), three to branched chain fatty acids (BCFA), four to hydroxyl saturated fatty acids (OH-SFA) and three to 2-hydroxy branched chain fatty acids (2OH-BCFA). Qualitatively, the greatest number of different FA were detected in the milk fermented with *Lb. paracasei* ABK (16—SFA, six—MUFA, one—PUFA, one—BCFA, four—OH-SFA and three—2OH-BCFA), followed by the milk fermented by *Lc. lactis* AM1 (18—SFA, seven—MUFA, two—PUFA and three—2OH-BCFA), *Lb. delbrueckii* Lb100 (14—SFA, five—MUFA, one—PUFA and two—2OH-BCFA) and *Str. termophilus* 159 (13—SFA, seven—MUFA, two—PUFA, three—BCFA). In terms of overall FA contents, in the milk fermented with *Lb. delbrueckii* Lb100 the proportion of MUFA and 2OH-BCFA (23% and 2.7%, respectively) was significantly higher than in other fermentation products. The BCFA was totally absent in the milk fermented with *Lb. paracasei* ABK and *Lc. lactis* AM1, and in the milk fermented with *Str. termophilus* 159 their content was significantly higher than that in the milk fermented by *Lb. paracasei* ABK (1.9 vs. 0.06%,). The OH-SFA were detected only in the milk fermented with *Lb. paracasei* ABK (2.7%) and *n*-3 PUFA only in the milk fermented with *Lc. lactis* AM1 (0.3%). The proportion of SFA was statistically the same for all fermentation products (approximately 65%).

It is well known that the consumption of certain dietary fats, which are generally FA, may exert either positive or negative effects on human health [40,41,42]. To evaluate the potential role of FA content of food in the treatment and prevention of cardiovascular diseases, several FA-related nutritional indices have been developed. Currently, the most frequently used FA-related nutritional indices are [32]: PUFA/SFA—ratio of total PUFA to total SFA; IA—index of atherogenicity; HPI—health-promoting index (which is the reciprocal of IA and mainly used in research on dairy products); IT—index of thrombogenicity; HH—hypocholesterolemic/hypercholesterolemic ratio; UI—unsaturation index. The calculation of mentioned indices for the studied dairy beverages as well as their general interpretation are presented in Table 3. Generally, all of the calculated indices were in the range previously reported for different dairy products such as yogurt, kefir, ryazhenka and amasi [31,32]. The comparison of studied fermentation products in terms of the mentioned FA-related nutritional indices did not detect any statistically-significant differences (*p* > 0.05). The exception was IA, HPI and HH indices by which milk fermented with *Lb. delbrueckii* Lb100 significantly outperformed (*p* < 0.05) all other fermentation products.

### 3.3. The In Vivo Assessment of the Antihypertensive Properties for the Milk Fermented by the Selected LAB Strains in the SHR Animal Model

The *in vivo* assessment of the antihypertensive properties for the milk fermented by the chosen LAB strains was performed in SHR animal model. Currently, SHR is one of the most popular animal models of essential (or primary) hypertension [43,44,45]. The experimental setup included six groups of SHRs: one **Intact**, one **Control** and four experimental. The **Intact** animals received only a standard daily ration; the ration of the **Control** group was supplemented with milk; and the ration of the experimental groups **Lb100**, **ABK**, **AM1** and **St159** was supplemented with milk fermented by *Lb. bulgaricus* LB100, *Lb. paracasei* ABK, *L. lactis* AM1 and *Str. thermophilus* 159, respectively.

**Figure 2:**
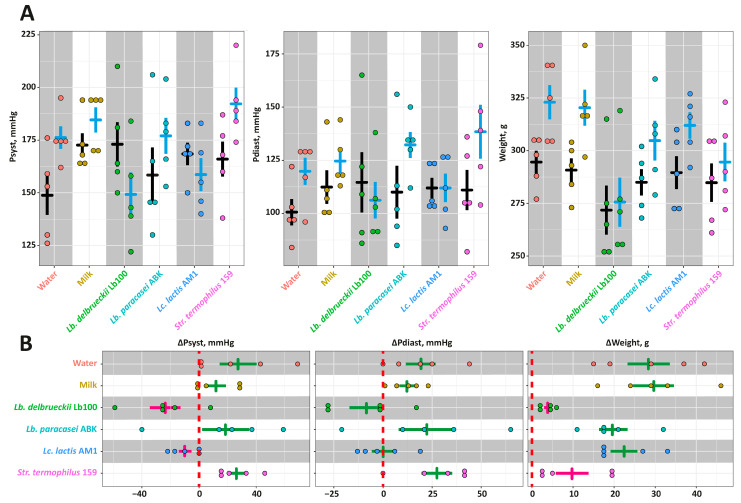
The *in vivo* assessment of antihypertensive properties for the milk fermented by the chosen strains of lactic acid bacteria using Spontaneously Hypertensive Rat (SHR) animal model: (**A**)—the absolute values of the Psyst, Pdias and Weight. The black bars correspond to the Mean ± SE at the beginning of the experiment, and the blue bar correspond to the Mean ± SE at the end (4 weeks) of the experiment; (**B**)—the changes of the Psyst, Pdias and Weight. The bars (Mean ± SE) for the groups between which no statistically-significant differences were detected are depicted in the same color.

The data on the absolute values of the Psyst, Pdias and Weight for each animal group at the beginning and end of the experiment are present in Figure 2A, and the changes of these parameters (ΔPsyst, ΔPdias and ΔWeight) are presented Figure 2B. For all animal groups but **Lb100** and **AM1**, Psyst increased at the end of the experiment and reached approximately 182 mmHg, and ΔPsyst was approximately 21 mmHg. For **Lb100** and **AM1**, the Psyst decreased down to 154 mmHg at the end of experiment, and ΔPsyst was approximately −17 mmHg. While no statistically-significant changes (*p* > 0.05) in Pdias for all studied groups were detected, for **Lb100** and **AM1** the downward trend in Pdias was observed. In case of the Weight, the was no statistically-significant difference (*p* > 0.05) between ΔWeight of the **Intact**, **Control**, **ABK**, and **AM1** groups. Animals from these groups gained on average 25 g at the end of the experiment, reaching the final weight of 315 g. In comparison, the weight gains of **Lb100** and **St159** were significantly smaller (*p* < 0.05) and comprised 7 g, reaching the final weight of 285 g.

**Figure 3:**
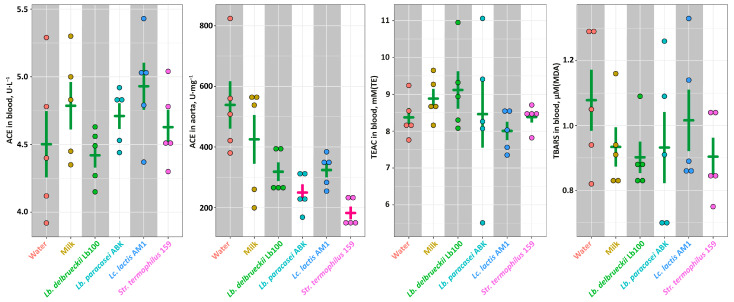
The ACE activity in blood and aorta, as well as the markers of the oxidant-antioxidant status for studied animal groups. The bars (Mean ± SE) for the groups between which no statistically-significant differences were detected are depicted in the same color.

At the end of the experiment all animals were euthanized, and several health-relevant biochemical parameters were assessed. No statistically-significant differences (*p* > 0.05) of the ACE activity were observed in the animals’ blood (Figure 3). In the animals’ aorta, the ACE activity was significantly lower (*p* < 0.05) in the **ABK** and **St159** groups compared to the **Intact** and **Control** groups, and for the **Lb100** and **AM1** groups only the downward trends were observed. For all animals, the level of the oxidative stress was assessed by the measuring of TEAC and TBARS concentration in blood (Figure 3). The TEAC value represent an overall antioxidant capacity of biological sample [46], and TBARS value corresponds to the advancement of lipid peroxidation under the oxidative stress. For all animal groups, the values of both TEAC and TBARS were not statistically-significantly different (*p* > 0.05). However, there was a trend for the milk fermented with *Lb. delbrueckii* Lb100 to have higher TEAC and lower TBARS than in other fermented products.

**Figure 4:**
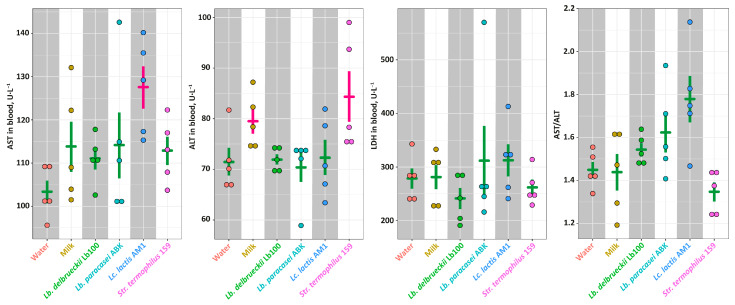
The markers of the liver health for studied animal groups. The bars (Mean ± SE) for the groups between which no statistically-significant differences were detected are depicted in the same color.

The function of liver was assessed by measuring widespread biomarkers of liver health—AST, ALT and LDH (Figure 4). Although the significantly elevated (*p* < 0.05) level of AST was detected in the **AM1** group, and the significantly elevated (*p* < 0.05) level of ALT in the **Milk** and **St159** groups, the AST/ALT ratios were not statistically-significantly different (*p* > 0.05) for all studied groups. Similarly, the level of LDH in blood was the same for all studied groups.

**Figure 5:**
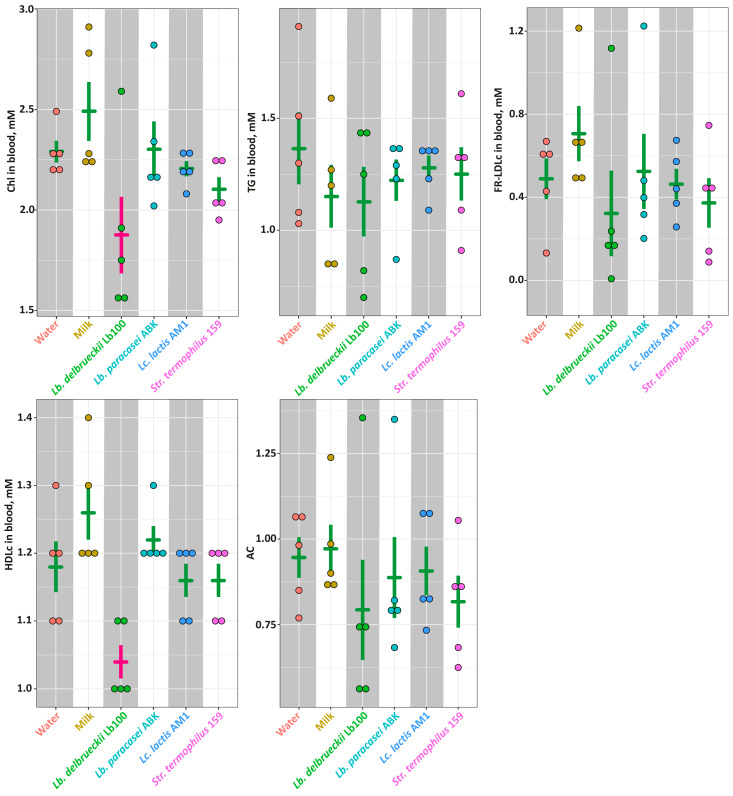
The markers of the lipid metabolism for studied animal groups. The bars (Mean ± SE) for the groups between which no statistically-significant differences were detected are depicted in the same color.

The lipid profile (i.e., Chl, TG, FR-LDLc and HDLc) for each animal group as well as the value of atherogenic coefficient (AC) calculated according to Nimmanapalli et al. [47] are presented in Figure 5. Among studied groups, there was no statistically-significant differences (*p* > 0.05) detected by all measured parameters. The exceptions were levels of Chl and HDLc that were significantly lower (*p* < 0.05) in the **Lb100** group.

## 4. Discussion

Hypertension is a serious medical condition that significantly increases the risks of cardiovascular and other diseases [48]. The extreme widespread of hypertension in modern world forces scientists to look for new ways of its prophylaxis, among which antihypertensive functional food, especially fermented dairy products, is the most promising one [49]. Therefore, the objective of the current study was to compare different strains of LAB commonly used in dairy industry on their ability to produce fermented milk with antihypertensive properties. At the first stage of the investigation, the *in vitro* assessment of ACE-I properties of milk fermented by different LAB strains was carried out, and the best performed strains were selected. At the second stage, the antihypertensive properties of the milk fermented by the selected strains were tested *in vivo* using SHR animal model. Additionally, the FA composition of the fermented milk was investigated, and FA-related nutritional indices linked with cardiovascular health were calculated.

At the first stage of the investigation, two strains of *Lb. delbrueckii* (Lb100 and Lb200), three strains of *Lc. lactis* (dlA, AM1 and MA1), two strains of *Str. thermophilus* (159 and 16t) and the strain *Lb. paracasei* ABK were tested (Figure 1). As a result, the strains *Lb. bulgaricus* Lb100, *Lc. lactis* AM1, *Str. thermophilus* 159, and *Lb. paracasei* ABK demonstrated ACE IC_50_ of 1–1.5 mg·mL^−1^, 1.8–2.0 mg·mL^−1^, 1–1.5 mg·mL^−1^, and 2.1–2.5 mg·mL^−1^, respectively, were chosen for further investigations. Currently, milks fermented with *Lactobacillus* spp. are the most commonly studied for ACE-I activity, while data about *Lactococcus* spp. and *Streptococcus* spp. are relatively scarce. The typically reported diapason of IC_50_ for the different strains of *Lb. bulgaricus* (and for *Lactobacillus* spp. in general) is 0.5–10 mg·mL^−1^ [9,19,50,51,52,53]. The typically reported diapason of IC_50_ for the different strains of *Lc. lactis* is 0.1–8 mg·mL^−1^ [51,52,53,54,55], and for the different strains of *Str. thermophilus* is 0.2–0.8 mg·mL^−1^ [50,51,52]. Thus, although our strains did not show outstanding results in terms of IC_50_ of fermented milk, they demonstrated values close to the lower end of the previously reported range.

At the second stage of the investigation, SHRs were fed by milk fermented with the selected LAB strains and changes in systolic and diastolic blood pressure were measured. Additionally, several biochemically relevant parameters such as: ACE activity in aorta; ACE, AST, ALT and LDH activities in blood; TEAC, TBARS, Chl, TG, HDLc and FR-LDLc concentration in blood—were assessed. Out of the four selected fermentation products, which demonstrated substantial ACE-I activity *in vitro*, only the milks fermented by *Lb. bulgaricus* Lb100 and *Lc. lactis* AM1 demonstrated pronounced *in vivo* antihypertensive effect (Figure 2). While the consumption of the milk fermented with *Lc. lactis* AM1 stabilized Psyst of SHRs at the initial (at the beginning of the experiment) level, the consumption of the milk fermented with *Lb. bulgaricus* Lb100 lowered Psyst of SHRs during the experiment. The observed poor agreement between *in vitro* (using the ACE-I assay) and *in vivo* (using appropriate animal models) methods for evaluation of antihypertensive properties of fermented milk is well known. The main reason of this poor agreement is that *in vitro* tests reflect only the interaction of the inhibitors (e.g., peptides) with ACE, and *in vivo* tests account for many other possible physiological factors involved in the manifestation of hypertension [20].

Comparing of our SHR experiments with previously published, it should be noted that most of the works are devoted to either products obtained using combined starter cultures [56,57,58,59,60] or products artificially enriched with antihypertensive peptides [61,62], and only few studies have tested hypotensive effect of milk fermented with individual strains. Previously, the *in vivo* effect of long-term intake of milk fermented with individual LAB strains was studied for the milk fermented with *Lc. lactis* [63,64], *Lactobacillus helveticus* [60,61,65], *Lb. delbrueckii* [63], and *Str. thermophilus* [63]. In most cases, the blood pressure of SHRs gradually increased during the experiment, and fermented milk only slowed down the development of hypertension (i.e., the Psyst at the end of experiments was higher than at their beginning). Only two studies, as well as our results, demonstrated a reduction in pressure not only relative to the control but also relative to the starting point of the experiment [58,65].

To find out whether the observed *in vivo* antihypertensive effect is the result of ACE inhibition, we measured the ACE activity in the blood and aorta of SHRs (Figure 3). While ACE activity in the blood was not statistically-significantly different (*p* > 0.05) for all studied groups, the decrease of the ACE activity in the aorta of SHRs consuming fermented milk products was detected. This is consistent with the data of other researchers – in the works of Nakamura et al. [56] and Kim et al. [57], when animals were fed with hypotensive fermented milk, the ACE activity in the blood plasma did not change, while the ACE activity in the aorta was significantly reduced. However, consumption of milks fermented with *Lb. paracasei* ABK and *Str. thermophilus* 159 statistically-significantly reduced (*p* < 0.05) the activity of ACE in the aorta, but did not affect blood pressure (Figure 2 and Figure 3). At the same time, milks fermented with *Lb. bulgaricus* Lb100 and *L. lactis* AM1, for which only a trend (*p* > 0.05) for a decrease in ACE activity in the aorta was detected, caused significant (*p* < 0.05) improvement of Psyst (Figure 2 and Figure 3). This once again emphasizes that the development of primary hypertension is a complex process for which a causal relationship is not always clear.

It is generally recognized that hypertension is closely associated with endothelial dysfunction, which leads to a decrease in the bioavailability of nitric oxide, one of the main vasodilators in the human body, due to an increased level of oxidative stress (nitric oxide reacts with the reactive oxygen species, ROS). In addition, several cross-sectional studies suggested a link between dyslipidemia and hypertension, and proposed that dyslipidemia can cause endothelial damage [66,67,68,69]. Hypertension occurs more frequently for hypercholesterolemic subjects, as compared to normolipid [70].

In our work, we evaluated the level of oxidative stress by measuring TEAC and TBARS in the blood, as well as the blood lipid profile (Figure 3 and Figure 5). Unfortunately, at this stage, we cannot put forward any hypotheses about the reasons for the decrease in Psyst upon the consumption of milk fermented with *Lc. lactis* AM1. However, the effect of reducing blood pressure upon the consumption of milk fermented with *Lb. bulgaricus* Lb100 can be related to its two additional to ACE-I properties—antioxidant and hypocholesterolemic. Compared to other animal groups, there was a strong trend for SHRs consuming the milk fermented with *Lb. bulgaricus* Lb100 to have increased level of TEAC and decreased level of TBARS in the blood, which suggests their better oxidant/antioxidant status. Moreover, the milk fermented with *Lb. bulgaricus* Lb100 statistically-significantly decreased the level of blood cholesterol (*p* < 0.05).

Since the consumption of certain FAs was associated with the risks of cardiovascular and other diseases for several decades [71,72,73,74], for each *in vivo* tested fermented milk the analysis of FA profile was performed. It is generally accepted that substitution of SFA with *n*-3 PUFAs and MUFAs in diet can exert positive cardiovascular effects via improvement of plasma membrane fluidity, decreasing the risk of inflammation, improving oxidant/antioxidant status etc. [75]. Currently, several FA-related nutritional indices that take into account both types of SFAs and their ratio to *n*-3 PUFAs and MUFAs in different ways (Equations (1)–(6)) have been developed; however, the potential usage of these indices in disease prevention and treatment is still a controversial issue [32]. Interestingly, the milk fermented with *Lb. bulgaricus* Lb100 demonstrated a number of improved FA-related nutritional indices (i.e., IA, HPI and HH) compared to other products (Table 2). Hence, our results suggested that at least in case of fermented dairy products such FA-related nutritional indices as IA, HPI and HH can have a potential predictive value for prevention of primary hypertension.

## 5. Conclusions

In the present study, the eight dairy products obtained via the fermentation with different starter strains of LAB were tested *in vitro* for their ACE-I activity, and four best performed products were further tested *in vivo* in SHR animal model for their antihypertensive effect. Only two out of four products demonstrated pronounced *in vivo* antihypertensive effect, which highlights the poor agreement between the *in vitro* (using the ACE-I assay) and *in vivo* (using SHR animal model) methods for evaluation of antihypertensive properties of fermented milk. Moreover, the most prominent *in vivo* antihypertensive effect was demonstrated for the milk fermented with *Lb. bulgaricus* Lb100, consumption of which did not significantly reduced (*p* > 0.05) the ACE activity in the aorta of SHRs (although, the trend for its reduction was observed), suggesting a more complex reason of its antihypertensive action than just ACE inhibition. It was further shown that consumption of milk fermented by *Lb. bulgaricus* Lb100 significantly (*p* < 0.05) reduced the level of total cholesterol in the blood of SHRs, and improved (as a trend, *p* > 0.05) their oxidant/antioxidant status (i.e., increased level of TEAC and decreased level of TBARS in their blood). It can be hypothesized that the pronounced antihypertensive effect of the milk fermented with *Lb. bulgaricus* Lb100 can be a result of synergy between decrease of ACE activity and improvement of endothelial functions via lowering of cholesterol and improvement of oxidant/antioxidant status. Additionally, the milk fermented with *Lb. bulgaricus* Lb100 demonstrated a number of improved FA-related nutritional indices (i.e., IA, HPI and HH) that were previously linked with cardiovascular health, although their predictive value is still debatable. Therefore, *Lb. bulgaricus* Lb100 can be regarded as promising starter strain for production of functional fermented milk or yogurt with antihypertensive and hypocholesterolemic properties without additional ingredients in its recipe, although the exact mechanism of its health promoting action is still remains to be discovered.

## Figures and Tables

**Table 1:** Strains of lactic acid bacteria that were used in this study.

Strain	16S rRNA GeneBank Accession	Optimal Growth Temperature
***Lactobacillus delbrueckii***		
Lb100	MN994622	37 °C
Lb200	MN994623	
***Lacticaseibacillus paracasei***		
ABK	MN994625	30 °C
***Lactococcus lactis***		
dlA	MN994624	30 °C
AM1	MW558124	
MA1	MW558123	
***Streptococcus thermophilus***		
159	MN994626	37 °C
16t	MN994627	

**Table 2:** Fatty acids (FA) composition of the milk fermented by the chosen strains of lactic acid bacteria.

Fatty Acid		Relative Abundance, %							
Name	Abbreviation	*Lb. delbrueckii* Lb100		*Lb. paracasei* ABK		*Lc. lactis* AM1		*Str. termophilus* 159	
		Mean	SD	Mean	SD	Mean	SD	Mean	SD
**Saturated Fatty Acids (SFA)**									
Pentanoic acid	C5:0	ND	-	0.03	0.01	0.03	0.01	ND	-
Hexanoic acid	C6:0	5.06 ^a^	0.32	2.99 ^b^	0.14	3.29 ^b^	0.23	3.42 ^b^	0.13
Heptanoic acid	C7:0	0.16	0.08	0.09	0.13	0.11	0.03	ND	-
Octanoic acid	C8:0	8.09 ^a^	0.16	4.84 ^b^	0.17	5.53 ^b^	0.39	6.38 ^b^	0.09
Nonanoic acid	C9:0	0.32	0.03	0.28	0.03	0.22	0.08	0.24	0.08
Decanoic acid	C10:0	10.36 ^a^	1.12	6.49 ^b^	1.01	7.43 ^b^	0.91	8.11 ^b^	1.80
Undecanoic acid	C11:0	ND	-	0.17	0.07	0.23	0.05	0.21	0.15
Dodecanoic acid	C12:0	4.01 ^a^	0.95	6.06 ^b^	0.14	6.82 ^b^	0.23	6.54 ^b^	0.43
Tridecanoic acid	C13:0			0.30	0.22	0.27	0.02	0.23	0.03
Tetradecanoic acid	C14:0	6.15 ^a^	1.30	9.64 ^b^	0.42	8.82 ^b^	1.01	8.90 ^b^	0.18
Pentadecanoic acid	C15:0	1.42	0.52	2.07	0.68	2.10	0.23	2.16	0.25
Hexadecanoic acid	C16:0	15.96	1.80	14.77	4.23	15.52	3.20	16.24	4.01
Heptadecanoic acid	C17:0	ND	-	1.21	0.11	1.07	0.08	0.91	0.12
Octadecanoic acid	C18:0	13.29	3.12	13.18	5.20	12.75	0.74	12.69	3.25
Eicosanoic acid	C20:0	0.61 ^a^	0.18	ND	-	1.17 ^b^	0.01	0.88 ^a^	0.11
Docosanoic acid	C22:0	1.09 ^a^	0.06	0.61 ^b^	0.14	0.64 ^b^	0.22	ND	-
Tricosanoic acid	C23:0	0.78 ^a^	0.13	ND	-	0.44 ^b^	0.22	ND	-
Tetracosanoic acid	C24:0	0.72 ^a^	0.07	0.28 ^b^	0.08	0.30 ^b^	0.08	ND	-
**Total SFA**		68.05	4.16	63.02	6.84	66.77	3.61	66.91	5.50
**Monounsaturated fatty acids (MUFA)**									
4-Decenoic acid	C10:1 (*n*-6)	2.45 ^a^	0.33	1.48 ^b^	0.21	1.81 ^b^	0.41	1.99 ^b^	0.22
Dodecenoic acid	C12:1 (*n*-10)	ND	-	0.36	0.06	0.41	0.17	0.42	0.20
9-Tetradecenoic acid	C14:1 (*n*-5)	0.41 ^a^	0.09	1.75 ^b^	0.73	1.13 ^b^	0.24	0.91 ^b^	0.11
9-Hexadecenoic acid	C16:1 (*n*-7)	1.41	0.25	3.64	1.03	2.45	0.38	2.08	0.23
9-Octadecenoic acid	C18:1 (*n*-9)	16.07	1.98	12.45	2.23	13.63	3.01	17.67	2.35
11-Octadecenoic acid	C18:1 (*n*-7)	2.91 ^a^	0.63	9.19 ^b^	1.02	5.62 ^a^	2.01	3.59 ^a^	0.98
11-Eicosenoic acid	C20:1 (*n*-9)	ND	-	ND	-	3.25 ^a^	1.02	0.78 ^b^	0.09
**Total MUFA**		23.25 ^a^	2.12	28.87 ^b^	2.77	28.28 ^b^	3.81	27.44 ^b^	2.58
**Polyunsaturated fatty acids (PUFA)**									
9,12-Octadecadienoic acid	C18:2 (*n*-6)	5.99 ^a^	1.22	4.17 ^b^	2.03	4.14 ^b^	1.56	3.35 ^b^	0.69
5,8,11,14-Eicosatetraenoic acid	C20:4 (*n*-6)	ND	-	ND	-	ND	-	0.41	0.10
4,7,10,13,16,19-Docosahexaenoic acid	C22:6 (*n*-3)	ND	-	ND	-	0.33	0.05	ND	-
**Total PUFA**		5.99	1.22	4.17	2.03	4.48	1.56	3.77	0.70
**Branched chain fatty acids (BCFA)**									
Tetradecanoic acid, 9-methyl	9Me-C14:0	ND	-	0.63	0.11	ND	-	0.76	0.11
Hexadecanoic acid, 15-methyl-	15MeC16:0 (iso-C17:0)	ND	-	ND	-	ND	-	0.46	0.15
Hexadecanoic acid, 14-methyl-	14MeC16:0 (anteiso-C17:0)	ND	-	ND	-	ND	-	0.68	0.09
**Total BCFA**		ND	-	0.63 ^a^	0.11	ND	-	1.89 ^b^	0.21
**Hydroxy saturated fatty acids (OH-SFA)**									
Octanoic acid, 3-hydroxy-	3OH-C8:0	ND	-	0.05	0.20	ND	-	ND	-
Decanoic acid, 3-hydroxy-	3OH-C10:0	ND	-	0.07	0.22	ND	-	ND	-
Tetradecanoic acid, 3-hydroxy-	3OH-C14:0	ND	-	0.22	0.11	ND	-	ND	-
Octadecanoic acid, 10-hydroxy-	10OH-C18:0	ND	-	2.39	1.03	ND	-	ND	-
**Total OH-SFA**		ND	-	2.73	1.08	ND	-	ND	-
**2-hydroxy branched chain fatty acids (2OH-BCFA)**									
Butyric acid, 2-hydroxy-3-methyl-	2OH-3MeC4:0 (2OH-iso-C5:0)	ND	-	0.09	0.10	0.10	0.08	ND	-
Pentanoic acid, 2-hydroxy-4-methyl-	2OH-4MeC5:0 (2OH-iso-C6:0)	1.80 ^a^	0.32	0.43 ^b^	0.13	0.20 ^b^	0.26	ND	-
Pentanoic acid, 2-hydroxy-3-methyl-	2OH-3MeC5:0 (2OH-anteiso-C6:0)	0.91 ^a^	0.26	0.06 ^b^	0.02	0.18 ^b^	0.03	ND	-
**Total 2OH-BCFA**		2.72 ^a^	0.41	0.58 ^b^	0.17	0.48 ^b^	0.10	ND	-

ND: not detected; SD: standard deviation. ^a,b^ Means within the same row with different superscripts are significantly different (*p* < 0.05).

**Table 3:** Fatty acid (FA) related nutritional indices of the milk fermented by the chosen strains of lactic acid bacteria.

Index	General Interpretation	*Lb. delbrueckii* Lb100		*Lb. paracasei* ABK		*Lc. lactis* AM1		*Str. termophilus* 159	
		Mean	SD	Mean	SD	Mean	SD	Mean	SD
PUFA/SFA	The higher—The better	0.09	0.02	0.07	0.03	0.07	0.02	0.06	0.01
IA	The lower—The better	1.53 ^a^	0.15	1.80 ^b^	0.23	1.76 ^b^	0.24	1.87 ^b^	0.21
HPI	The higher—The better	0.66 ^a^	0.07	0.56 ^b^	0.07	0.57 ^b^	0.08	0.53 ^b^	0.06
IT	The lower—The better	2.42	0.20	2.28	0.27	2.15	0.26	2.42	0.22
HH	The higher—The better	0.84 ^a^	0.12	0.55 ^b^	0.12	0.58 ^b^	0.11	0.68 ^b^	0.09
UI	The higher—The better	35.22	2.45	37.21	3.43	38.56	4.12	35.79	2.67

ND: not detected; SD: standard deviation. ^a,b^ Means within the same row with different superscripts are significantly different (*p* < 0.05).

## Data Availability

Not applicable.
